# Piperacillin-Tazobactam Induced Rapid Severe Thrombocytopenia Without Known Exposure

**DOI:** 10.7759/cureus.26970

**Published:** 2022-07-18

**Authors:** Amrit Bhaskarla, Mateusz D Gorecki, Masood Ghouse

**Affiliations:** 1 Hematology and Medical Oncology, Franciscan Health Olympia Fields, Olympia Fields, USA; 2 Internal Medicine, Franciscan Health Olympia Fields, Olympia Fields, USA

**Keywords:** beta lactam, piperacillin, thrombocytopenia, drug induced thrombocytopenia, piperacillin-tazobactam

## Abstract

Thrombocytopenia is one of the commonly encountered laboratory abnormalities in the inpatient setting. The process of excluding life-threatening causes can be daunting and may result in overlooking iatrogenic sources such as medications. Antibiotics are known culprits; however, there are limited reports of rapid and severe onset thrombocytopenia following piperacillin-tazobactam (TZP) that were frequently observed in critically ill or immunocompromised patients with previous exposure to the antibiotic. This case describes a patient being treated for a soft tissue infection with vancomycin and TZP. Initiation of antimicrobial therapy resulted in severe thrombocytopenia and a platelet nadir of approximately 4,000 within 24 hours of the first doses. Thrombocytopenia resolved within three days of TZP withdrawal. To the best of our knowledge, there have not been any cases described of rapid drug-induced thrombocytopenia without previous exposure to the medication. Medications should always be reviewed when evaluating a patient with rapid and severe thrombocytopenia, which can obviate the need for unnecessary invasive or non-invasive treatments.

## Introduction

Thrombocytopenia is frequently identified in hospitalized patients, especially in patients admitted to the intensive care unit, with an incidence between 13 and 44% [1]. Piperacillin-tazobactam (TZP) is a beta-lactam antibiotic that is one of the most commonly used antimicrobials in the hospital setting [2]. This antibiotic can result in a multitude of adverse reactions, with thrombocytopenia seen in up to 37% of patients based on a review of case reports [3]. Drug-induced thrombocytopenia is a well-known phenomenon with a complex pathophysiology that differs for different medications, of which over 100 are implicated [4]. Vancomycin is more commonly associated with thrombocytopenia in clinical situations, but TZP is also an important and possibly overlooked cause [5]. One proposed mechanism of TZP causing platelet destruction is through drug-macromolecule linked ‘hapten’ antibodies resulting in induction of the humoral immune system and ensuing drug-induced immune thrombocytopenia (DITP) [4]. Platelet reactive antibodies can be potentially tested for confirmation of DITP [4,5]. Rapid onset thrombocytopenia due to medications can occur within one day if patients have been exposed to the same drug previously [6].

## Case presentation

A 54-year-old male with a history of type II diabetes mellitus, hidradenitis suppurativa, with recurrent bilateral inguinal and gluteal skin lesions presented to the emergency room with purulent drainage from a gluteal wound. There was no documented history of thrombocytopenia or known exposure to piperacillin-tazobactam, although the patient was admitted within the last six months for soft tissue infections for which he received vancomycin, ampicillin-sulbactam, and ceftriaxone.

**Figure 1:**
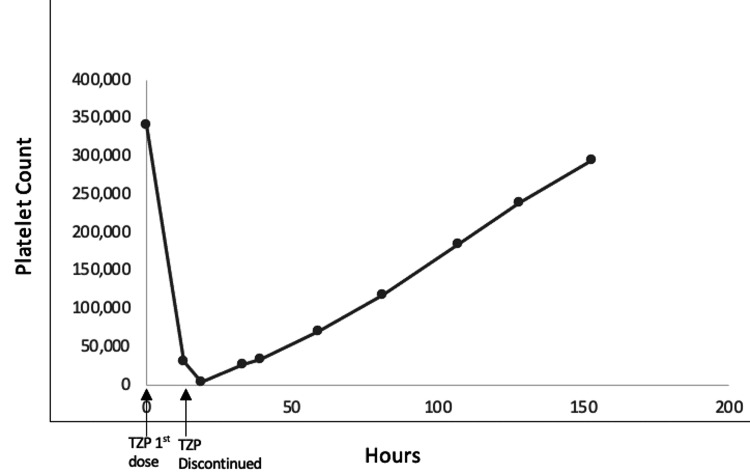
Platelet trend related to TZP TZP - piperacillin-tazobactam

Vitals signs were stable on admission. The hemoglobin was 9.5 g/dL, and the platelet count was 342,000/uL. The patient was admitted to the general medical floor and given one dose of intravenous (IV) TZP 3.375 g and two doses of IV vancomycin 1,250 mg as well as one dose of subcutaneous prophylactic enoxaparin 40 mg. Thirteen hours after the first dose of TZP, the platelet count dropped to 32,000/uL as shown in Figure 1. The platelet count further worsened to 4,000/uL with an accompanying drop in hemoglobin to 6.8 g/dL, following a surgical incision and drainage performed later the same day, which was complicated by postoperative bleeding. Vitamin B12 and folate levels were normal. Lactate dehydrogenase was normal with an absolute reticulocyte count of 30,330 that was not suggestive of microangiopathic hemolysis. There were no schistocytes seen on the peripheral smear, and the morphology of scant platelets was normal. 

One unit of platelets and one unit of red blood cells were transfused with an improvement of platelets to 27,000/uL and hemoglobin to 8.4 g/dL. TZP was discontinued, and the patient was switched to meropenem and vancomycin. Three days later, platelet counts improved to 185,000/uL and later to 295,000/uL. The patient was followed up post discharge with platelet counts remaining stable at over 200,000/uL.

## Discussion

Drug-induced immune thrombocytopenia (DITP) is a known entity that is often overlooked due to concerns of other life-threatening etiologies. Generally, thrombocytopenia is classified into different categories by the cause. These include decreased production, increased destruction, dilution, and sequestration [7]. Due to the significant mortality and morbidity associated with thrombotic microangiopathies and heparin-induced thrombocytopenia, these are frequently excluded upon initial examination of patients with low platelets [8]. Immune thrombocytopenia and malignant conditions of the bone marrow are also of concern when evaluating thrombocytopenia [8]. Further evaluation and treatment of these conditions may require invasive measures, including bone marrow biopsies, steroids, plasmapheresis, and intravenous immunoglobulin [8].

Thrombocytopenia is related to over a hundred medications, which act in different ways to decrease platelet counts [9]. Myelosuppression is one method of how drugs can decrease platelets by decreasing production from the bone marrow, with some medications even able to selectively suppress megakaryocyte production [9]. The other main mechanism of medications resulting in thrombocytopenia is a variety of immune-mediated processes [4]. One well-understood immune-mediated etiology is heparin-induced thrombocytopenia, where platelet factor 4 binds to heparin resulting in activation of platelets [4]. In contrast, piperacillin and other penicillin antibiotics are thought to work by linking to specific protein-forming units known as ‘haptens’ that induce humoral immune responses resulting in platelet destruction [4]. DITP usually occurs one to two weeks after the initiation of a medication but also may occur within hours in cases of re-exposure [10]. 

Hackett et al. previously proposed criteria to help with the diagnosis of DIPT as follows: 1) thrombocytopenia developed while the patient was taking the drug, resolved once the drug was stopped, and did not recur while the patient was off the drug; 2) other causes of thrombocytopenia were excluded; 3) thrombocytopenia recurred upon re-administration of the drug; 4) in-vitro test for drug-dependent antibodies was positive [10, 11].

Treatment of DITP is centered around discontinuation of the offending agent with an expected improvement of platelet counts within two days [10]. Platelet transfusions, intravenous immunoglobulin (IVIG), or steroids are also options to help improve platelet counts in the setting of persistent thrombocytopenia or bleeding [10]. Previous cases of drug-induced thrombocytopenia were successfully treated with combinations of these proposed treatments as well as no intervention [12].

Interestingly, the patient presented in this report experienced an acute and rapid severe thrombocytopenia following TZP exposure even though there was no documented history of prior TZP use. The patient was on an ampicillin-based antibiotic previously, which raises suspicion for possible cross-reactivity between the immune-mediated thrombocytopenia between penicillin-based antimicrobial agents. This was also considered in a previous report, given the structural similarities in beta-lactam antibiotics [13]. The patient also did not have persistent bleeding, so the withdrawal of TZP and platelet transfusions were adequate for maintaining clinical stability. Fortunately, the patient was switched to meropenem, which is an alternative beta-lactam antibiotic belonging to the carbapenem class [14].

## Conclusions

Drug-induced immune thrombocytopenia is an important entity that must be considered when elucidating the cause of acute thrombocytopenia. Antibiotics are known culprits and are one of the most used medications in the hospital. Recognition of DIPT is important as discontinuation of the offending agent may prevent further invasive diagnostic and therapeutic management of thrombocytopenia. Piperacillin-tazobactam may result in immune-mediated thrombocytopenia that can be potentially life-threatening and resolves when withdrawn. More research may be warranted to reveal if cross-reactive antibodies form between penicillin-based antibiotics, as considered in this case, given the rapid and severe thrombocytopenia seen with only previous known exposure to ampicillin. Documentation of DITP can help decrease exposures to the culprit agent during re-hospitalizations. Overall, DIPT recognition may not only aid in improving morbidity and mortality but also reduce associated healthcare costs. 
